# Supplementary material for: Phylogenetic inference enables reconstruction of a long-overlooked outbreak of almond leaf scorch disease (Xylella fastidiosa) in Europe
Source: Commun Biol. 2020 Oct 9;3:560. doi: 10.1038/s42003-020-01284-7 (PMC7547738; doi:10.1038/s42003-020-01284-7)
Supplement: Supplementary file 1 — Supplementary Information [file 42003_2020_1284_MOESM1_ESM.pdf]

Supplementary Information for

Phylogenetic inference enables reconstruction of a long-overlooked outbreak of almond leaf scorch disease (*Xylella fastidiosa*) in Europe

Eduardo Moralejo[1*], Margarita Gomila[2], Marina Montesinos[1], David Borràs[3], Aura Pascual[1], Alicia Nieto[3], Francesc Adrover[3], Pere A. Gost[4], Guillem Seguí[2], Antonio Busquets[2], José A. Jurado-Rivera[5], Bàrbara Quetglas[4], Juan de Dios García[4], Omar Beidas[4], Andreu Juan[4], María P. Velasco-Amo[6] Blanca B. Landa[6], Diego Olmo[3].

Eduardo Moralejo

Email: emoralejor@gmail.com

This PDF file includes:

Figures S1 to S9

Tables S1 to S5

SI References

Supplementary Figures

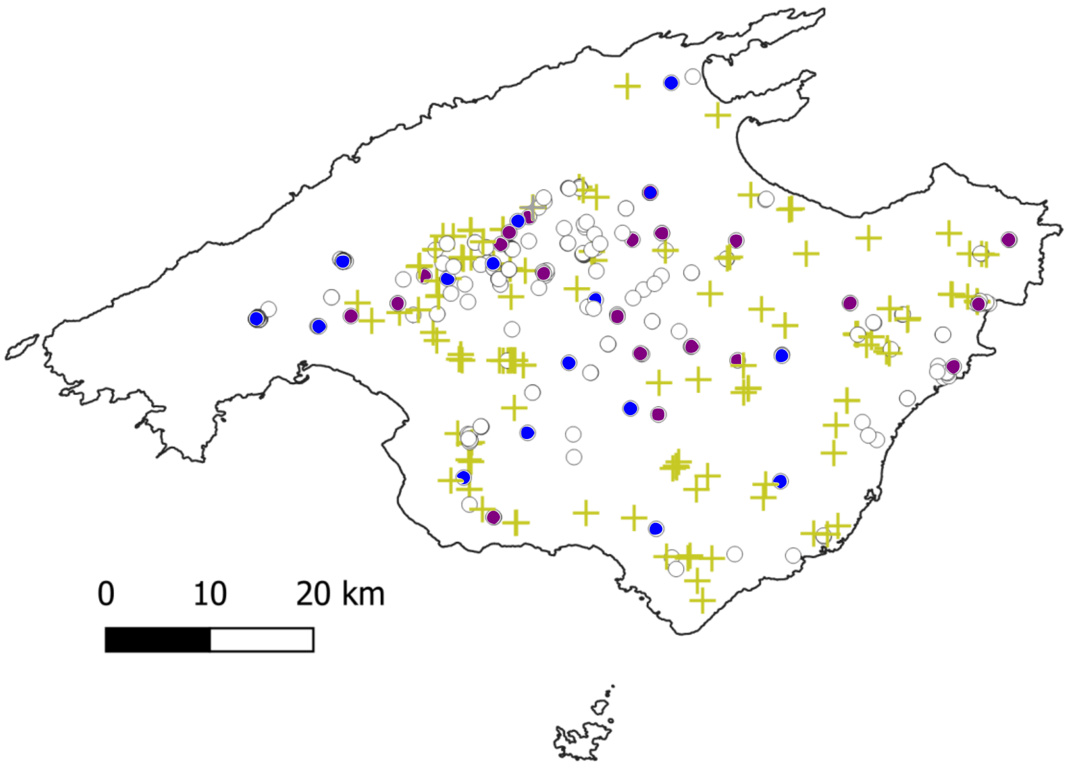

Supplementary Figure 1. Distribution map of almond (circles) and wild olive trees (green crosses) proven infected by qPCR for *Xylella fastidiosa* (*Xf*) in Majorca. All known wild olive tree infections (n = 84) are caused by *Xf* subsp. *multiplex* ST81, whereas almond infections (n = 119) are caused by *Xf* subsp. *fastidiosa* ST1 (purple filled circles) and subsp. *multiplex* ST81 (blue filled circles).

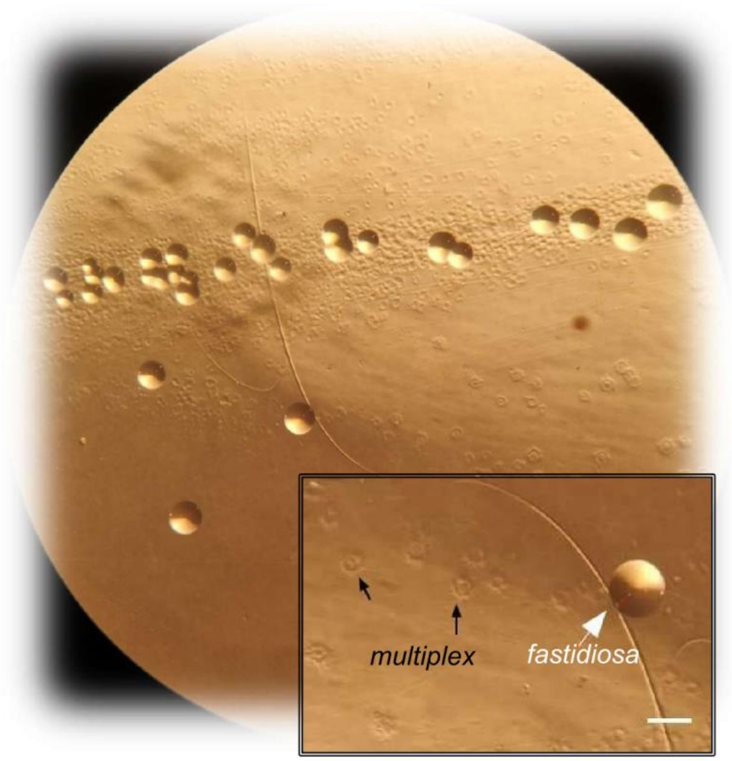

Supplementary Figure 2. Colony morphotypes[1] of *Xylella fastidiosa* (*Xf*) subsp. *fastidiosa* ST1 (G-type; white arrow) and *Xf* subsp. *multiplex* ST81 (A-type; black arrows) formed on periwinkle wilt GelRite (PWG) medium after streaking on a Petri dish the petiole extract from a single almond tree sample. The image was taken 15 days after plating, showing large differences in growing rates and colony morphologies (smooth type: *fastidiosa vs*. pit-like type: *multiplex*) between both strains. Scale bar = 1 mm.

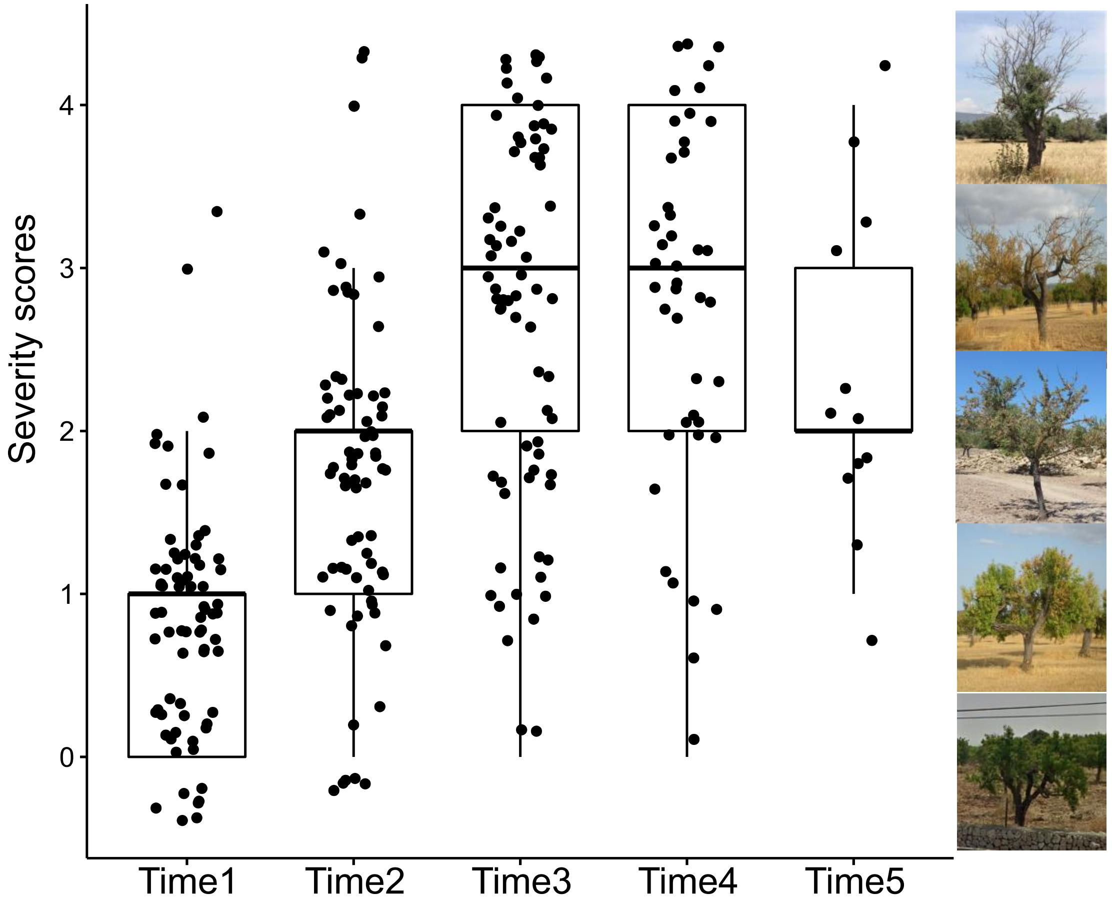

Supplementary Figure 3. Box plot showing the sequences of almond leaf scorch (ALSD) severity scores over time. Each point corresponds to the severity score of a single tree (*n* = 71) and its repeated measurements in an ordinal scale (Supplementary Data 3). On the right side of the figure images represent the symptom severity scale used (0 = healthy trees with no symptoms of ALSD; 1 = a branch or the whole canopy with leaf scorch symptoms; 2 = shoot and branch dieback affecting between 1 and 25% of the canopy; 3 = > 25-75% die-back; and 4 => 75% dieback or dead trees).

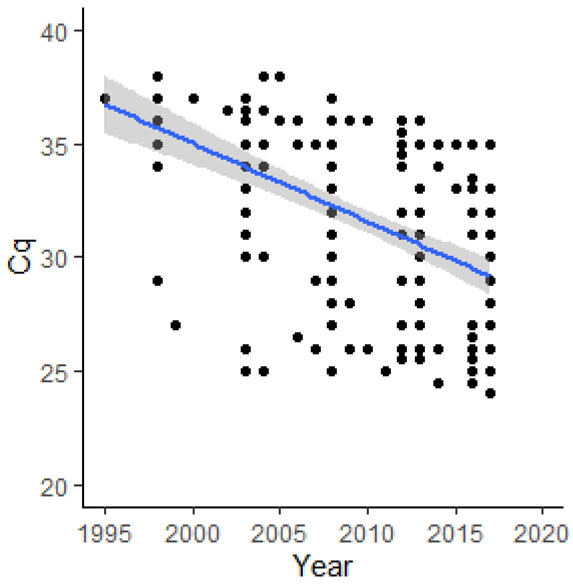

Supplementary Figure 4. *Xylella fastidiosa* (*Xf*) DNA concentration increases centrifugally from older to younger growth rings in almond wood sections. Threshold cycles (Cq) in the qPCR[2] assays were strongly correlated to the age of the growth ring from which the *Xf*-DNA was extracted across 34 trees (linear model: $F_{1,183}$ = 66.11, P = 0.0001). Lower Cq values in qPCR indicate higher concentrations of the target DNA.

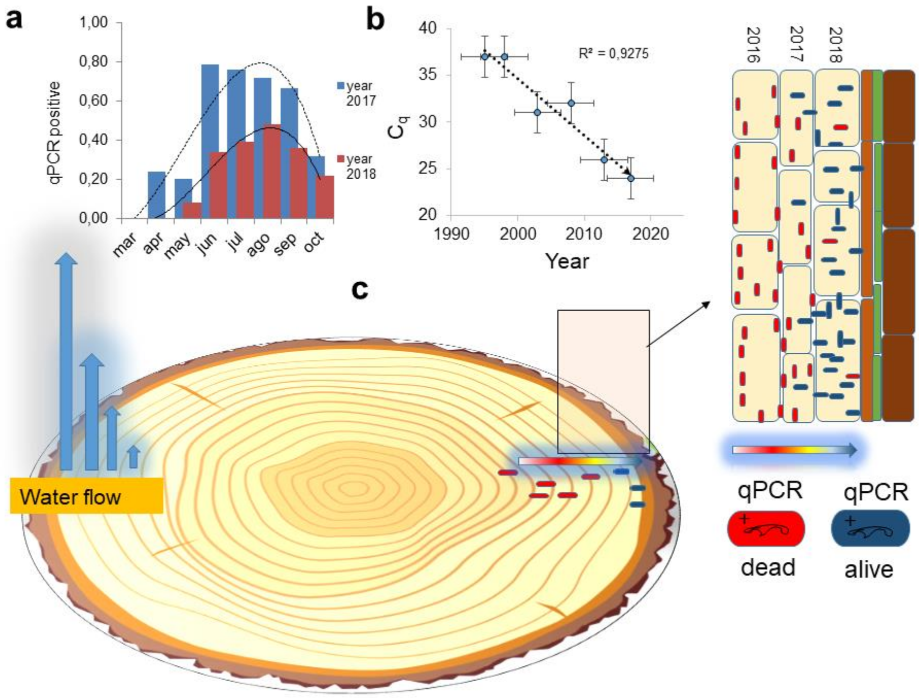

Supplementary Figure 5. Conceptual model explanation of *Xylella fastidiosa* (*Xf*) movement within the wood of infected almond trees. a) Trend of the annual colonization of xylem spring vessels in already infected trees obtained indirectly by calculating the frequency of almond samples that were qPCR[2] positives throughout the year. Every new spring *Xf* colonizes the newly formed xylem vessels towards the cambium accumulating bacterium load. This is reflected in the relationship between Cq and date of the tree rings **(b)**, which decreases from the pith to the cambium. **(c)** The bacterial directional movement is driven by the gradient of water flow and nutrients limited to the most external rings. *Xf* DNA is detected both in dead (red rods) and alive cells (blue rods) by qPCR but only living cells colonize the peripheral youngest rings.

.

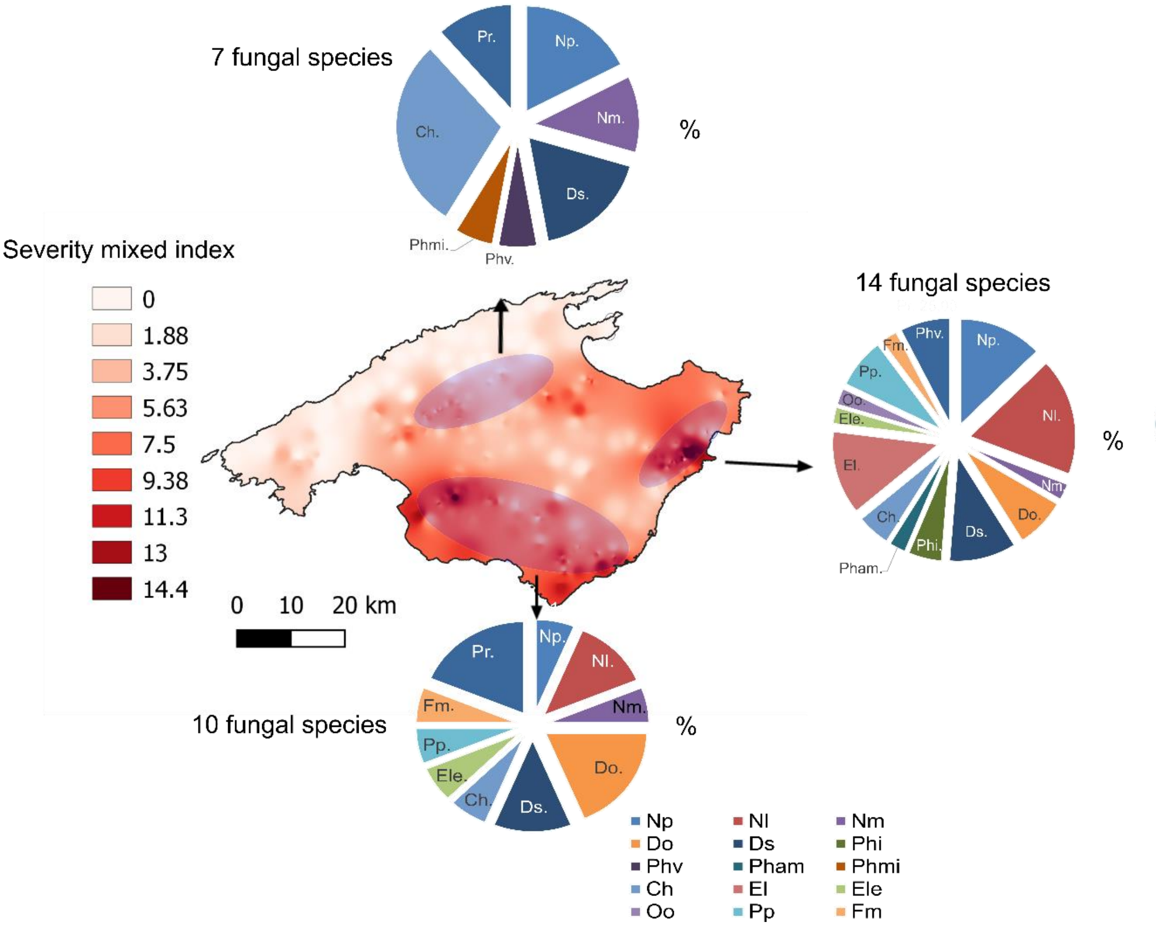

Supplementary Figure 6. Diversity of fungal trunk pathogens and their distribution related to almond leaf scorch disease severity in Majorca in 2012 after Olmo *et al*.[3]. Areas with longer exposure time to *Xylella fastidiosa* (east Majorca, Son Carrió) show greater fungal diversity compared to orchards in the south and centre of the island. Coastal areas in the east and south of Mallorca receive less annual precipitation and have shallow soils. Colour legend=severity index. Ch=*Collophora hispanica*; Do=*Diplodia olivarum*; Ds=*Diplodia seriata*; El=*Eutypa lata*; Ele= *Eutypa leptoplaca*; Fm=*Fomitiporia mediterranea*; Nl= *Neofusicoccum luteum*; Nm=*Neofusicoccum mediterraneum*; Np= *Neofusicoccum* Phv=*Phaeoacremonium venezuelense*; Pp=*Phellinus pomaceus*; Pr=*Pleurostomorpha richardsiae*.

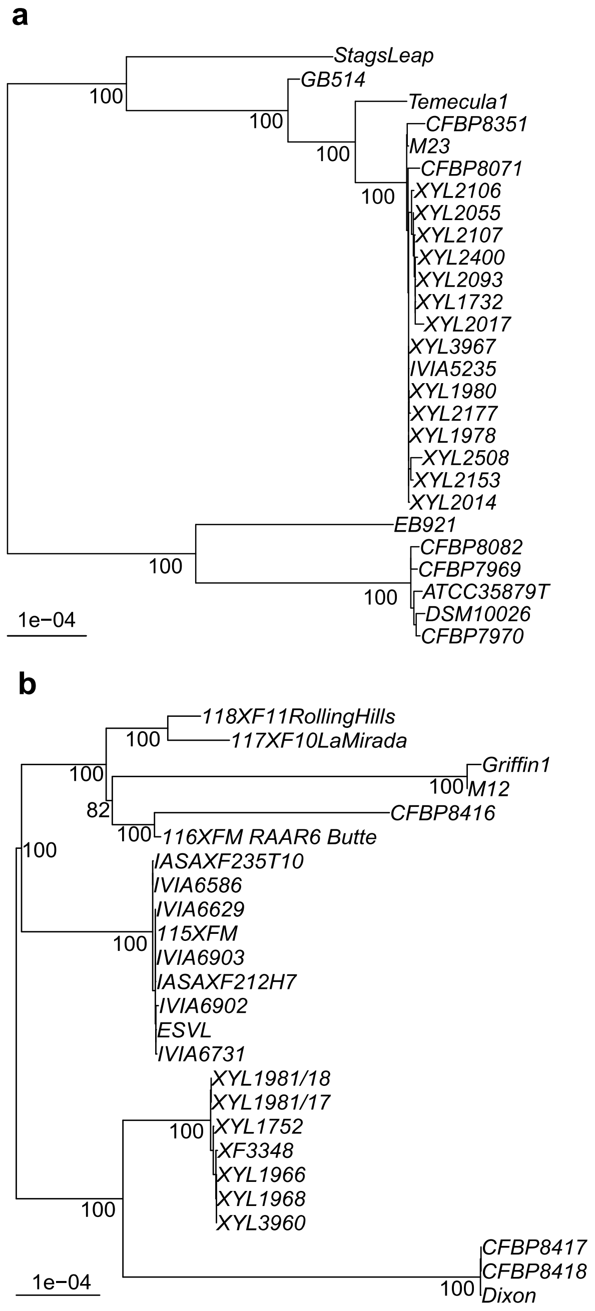

Supplementary Figure 7. Maximum likelihood (ML) phylogeny estimated with phangorn[4] for *Xylella fastidiosa* (*Xf*) subsp. *fastidiosa* and subsp. *multiplex* genomes related to recent introductions into Europe. (a) ML phylogenetic tree for 27 genomes of *Xf* subsp. *fastidiosa* isolates from California, south-east USA and Majorca **(b)** ML tree for 25 genomes of *Xf* subsp. *multiplex* from (Majorca and Menorca), Corsica (France), Alicante (Spain) and California. In both phylogenetic trees the GTR +G+I was the best fit model (Likelihood Ratio Test) with 1000 bootstraps. The numbers provide the node support bootstrap values (1000 replications). Bar scales represent number of substitutions per site.

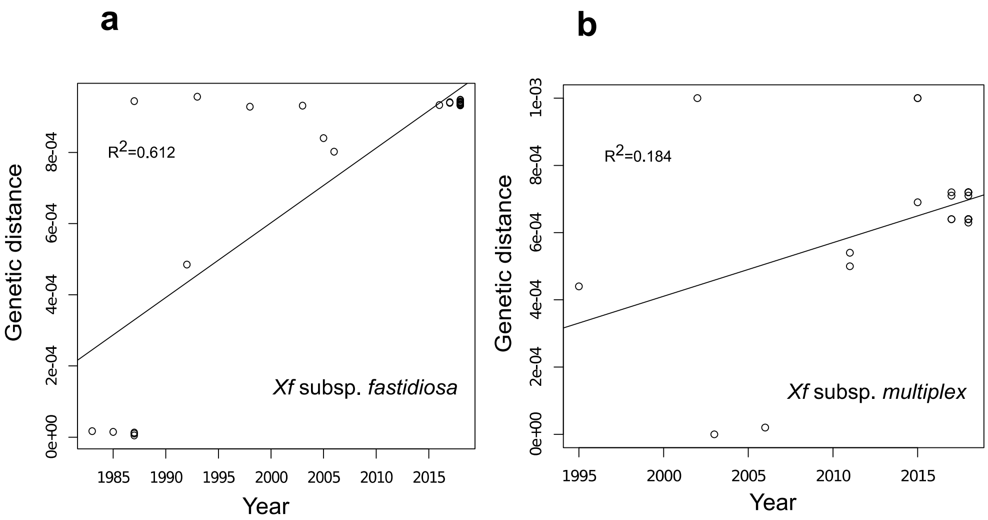

Supplementary Figure 8. Testing temporal signal for molecular clock calibration using tip dating. Linear regression between the age of the samples and their root-to-tip genetic distances for *Xylella fastidiosa* (*Xf*) subsp. *fastidiosa*. (a) from Mallorca (n = 15) and the USA (n = 12) ($F_{1,26}$ = 41.08, P < 0.0001) and *Xf* subsp. *multiplex* **(b)** from Majorca (n = 4), Menorca (n = 3), Alicante (Spain) (n = 9), Corsica (n = 3) and the USA (n = 6) ($F_{1,24}$ = 5.18, P = 0.0324). Root-to-tip distances were calculated in TempEst[5] using a Maximum Likelihood tree in PhyML[6].

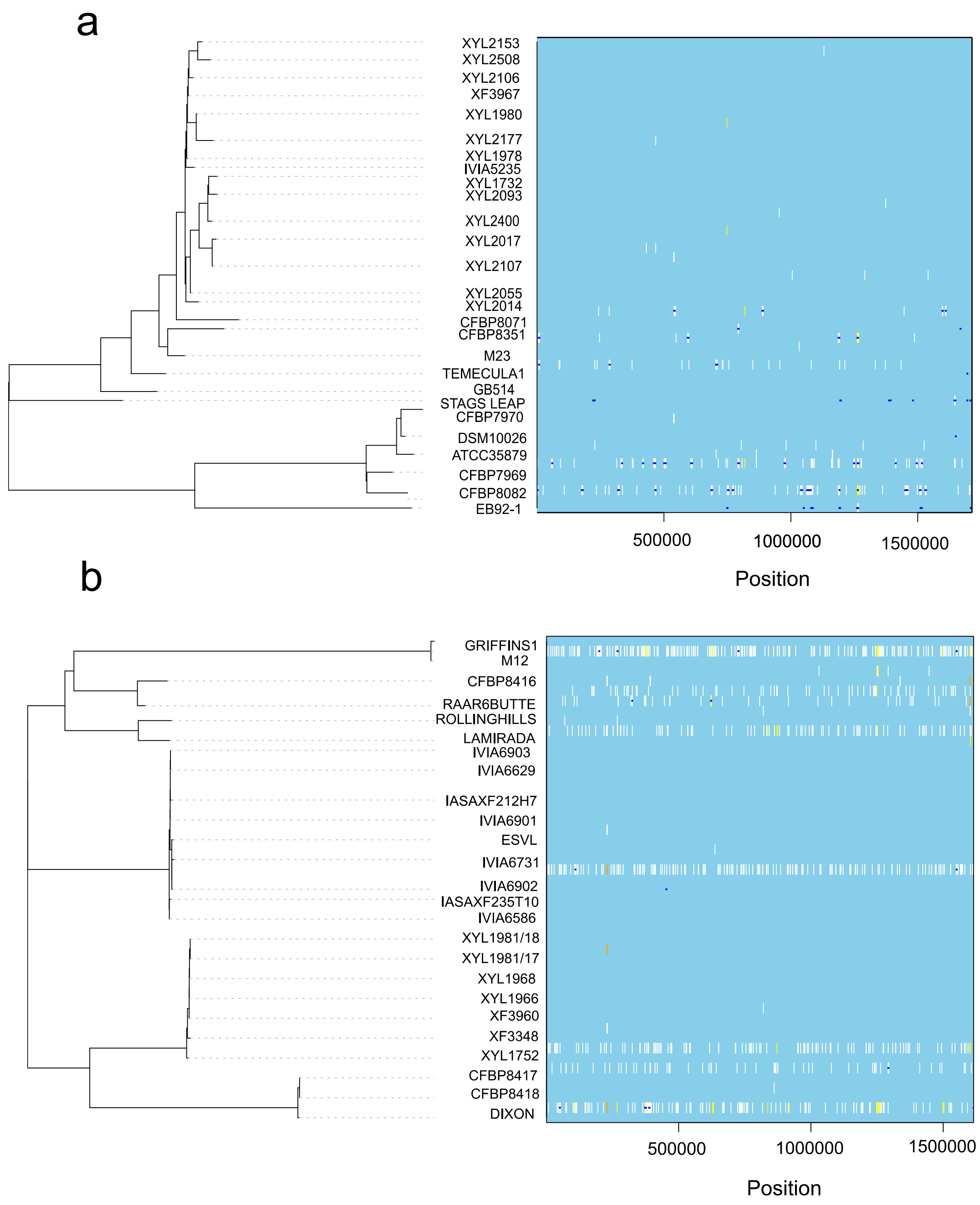

Supplementary Figure 9. Detection of homologous recombination using ClonalFrame. A panel **(a)** of 27 isolates of *Xylella fastidiosa* (*Xf*) subsp. *fastidiosa* ST1/ST2 from Majorca and the USA, and **(b)** 25 isolates of *Xf* subsp. *multiplex* (*n*=25) from Europe and the USA belonging to ST6/ST7 and ST81 were used.

Supplementary Tables

Supplementary Table 1. Susceptibility of non-local almond cultivars to *Xylella fastidiosa* (*Xf*) in the inoculation tests and in the field. Isolate XYL 2055/17 of *Xf* subsp. *fastidiosa* ST1 was used in the inoculations. Data from field infections were obtained from monitoring almond leaf scorch disease in four almond genetic bank collections in 2017 and 2018.

Almond cultivar	Rootstock	Infected %[a]	Field infection[b]
Marinada	G x N	0	Negative (0/14)
	GF 677	12.5	
Penta	G X N	0	Negative (0/6)
Avijor	G X N	0	NT
	GF 677	0	
	RP-20	25	
	RP-40	0	
Marta	GF 677	0	Negative (0/12)
Vairó	GF 677	12.5	Positive (1/14)
Soleta	GF 677	0	Negative (0/6)
	G x N	12.5	
	RP-20	50	
Belona	GF 677	12.5	Negative (0/13)
	G x N	12.5	
Vialfas	R-R	0	NT
	RP-20	25	
Pentacebas	G x N	75	NT
	RP-20	25	
Isabelona	R-20	12.5	NT
Guara	R-20	25	Positive (1/5)

[a] Percentage of *Xf* infections detected by qPCR[2] and isolation from eight inoculated plants 16 weeks after inoculation.

[b] Only the scion cultivar was considered as no information of rootstock (empty cells) was available; Negative= all plants tested qPCR-; Positive= at least one plant tested qPCR+; (number of qPCR positive/ number of samples tested); NT= Non tested.

Supplementary Table 2. Susceptibility of almond varieties (*n* = 110) to *Xylella fastidiosa* (*Xf*) infection in four almond germplasm collections under natural conditions. Leaf samples (*n* = 1061) were tested for *Xf* infection by qPCR[2,7] from late spring to late summer at the Plant Health Official Laboratory of the Balearic Islands.

Genebank Field	No. varieties	No. infected varieties (%)	No. non-infected varieties (%)	No. local varieties	No. non-local varieties	Plantation age
Fruit Secs	66	50 (74.2)	16 (25.8)	49	17	16
Xorrigo	14	3 (21.4)	11 (78.6)	0	14	5
Sa Canova	72	40 (55.5)	32 (44.5)	56	16	13
Son Real	77	52 (67.5)	25 (32.5)	66	11	6

Supplementary Table 3. Comparison of fungal assemblages found associated with the almond decline in Majorca, Spain and olive decline and mortality in Apulia, Italy.

Majorca<br>Olmo *et al*.[3]	Apulia, Gallipoli<br>Nigro *et al*.[8]	Apulia, Foggia<br>Carlucci *et al*.[9]
Neofusicoccum parvum	*Neofusicoccum parvum*	*Neofusicoccum parvum*
Pleurostomorpha richardsiae	*Pleurostomorpha richardsiae*	*Pleurostomorpha richardsiae*
Neofusicoccum luteum	*Neofusicoccum* spp.	*Neofusicoccum luteum*
Diplodia seriata	*Neofusicoccum vitifusiforme*	*Diplodia seriata*
Collophora hispanica	*Phaeoacremonium aleophilum*	*Phaeoacremonium aleophilum*
Neofusicoccum mediterraneum	*Neofusicoccum mediterraneum*	*Phaeoacremonium alevesii*
Fomitiporia mediterranea	*Phaeomoniella* spp.	*Fomitiporia mediterránea*
Neofusicoccum australe	*Neofusicoccum australe*	*Diplodia mutila*
Diplodia olivarum	*Phaeoacremonium rubrigenum*	*Botryosphaeria dothidea*
Phaeoacremonium iranicum	*Phaeoacremonium alvesii*	*Phaeoacremonium italicum*
Eutypa leptoplaca	*Phaeoacremonium parasiticum*	*Phaeoacremonium scolyti*
Phaeoacremonium amygdalinum		*Phaeoacremonium sicilianum*
Phomopsis amygdali		*Phaeoacremonium parasiticum*
Eutypa lata		*Lasiodiplodia theobromae*

Supplementary Table 4. Clock and tree model comparison results based on Bayes factor estimation through marginal likelihood estimation using both path-sampling and stepping-stone calculations in BEAST 1.10.4[10] for the 27 genomes of *Xylella fastidiosa* subsp. *fastidiosa* analysed. The selected model is highlighted in bold. All clock and tree model comparisons has been calculated using the GTR+I+R substitution model.

Clock Model	Tree model	Log marginal likelihood (using path sampling)	Bayes Factors (from path sampling)	Log marginal likelihood (using stepping stone sampling)	Bayes Factors (from stepping stone)
**Uncorrelated relax clock - Exponent**	**Coalescent: Bayesian Skyline**	**-2400832.4**	**0.00**	**-2400728.6**	**0.00**
Uncorrelated relaxed clock - Exponential	Coalescent: Constant size	-2400841.1	17.43	-2400833.7	210.25
Uncorrelated relaxed clock - LogNormal	Coalescent: Bayesian Skyline	-2400842.8	20.71	-2400836.9	216.67
Uncorrelated relaxed clock - LogNormal	Coalescent: Constant size	-2400857.9	50.98	-2400835.4	213.67
Strict clock	Coalescent: Bayesian Skyline	-2400983.7	302.61	-2400976.8	496.46
Strict clock	Coalescent: Constant size	-2400991.9	318.97	-2400984.6	512.00

Supplementary Table 5. Clock and tree model comparison results based on Bayes factor estimation through marginal likelihood estimation using both pathsampling and stepping-stone calculations in BEAST 1.10.4 for the 25 genomes of *Xylella fastidiosa* subsp. *multiplex* analysed. The selected model is highlighted in bold. All clock and tree model comparisons has been calculated using the HKY+I+R substitution model.

Clock Model	Tree Model	Log marginal likelihood (using path sampling)	Bayes Factors (from path sampling)	Log marginal likelihood (using stepping stone sampling)	Bayes Factors (from stepping stone)
**Uncorrelated relaxed clock - Exponential**	**Coalescent: Bayesian Skyline**	**-2265044.7**	**0.00**	**-2265037.4**	**0.00**
Uncorrelated relaxed clock - Exponential	Coalescent: Constant size	-2265063.5	37.55	-2265055.3	35.95
Uncorrelated relaxed clock - Lognormal	Coalescent: Bayesian Skyline	-2265050.7	11.96	-2265043.1	11.43
Uncorrelated relaxed clock - Lognormal	Coalescent: Constant size	-2265053.8	18.22	-2265047.1	19.33
Strict clock	Coalescent: Bayesian Skyline	-2265679.0	1268.57	-2265672.0	1269.22
Strict clock	Coalescent: Constant size	-2265708.3	1327.17	-2265700.7	1326.68

Supplementary References

1. Chen, J. *et al.* Two *Xylella fastidiosa* genotypes associated with almond leaf scorch disease on the same location in California. *Phytopathology* **95**, 708–714 (2005).
2. Harper, S. J., Ward, L. I. & Clover, G. R. G. Development of LAMP and Real-Time PCR Methods for the Rapid Detection of *Xylella fastidiosa* for Quarantine and Field Applications. *Phytopathology* **100**, 1282–1288 (2010).
3. Olmo, D. Etiología y control de las enfermedades fúngicas de la madera del almendro en la isla de Mallorca. (2016).
4. Schliep, K. P. phangorn: phylogenetic analysis in R. *Bioinformatics* **27**, 592–593 (2011).
5. Rambaut, A., Lam, T. T., Max Carvalho, L. & Pybus, O. G. Exploring the temporal structure of heterochronous sequences using TempEst (formerly Path-O-Gen). *Virus Evol.* **2**, vew007 (2016).
6. Guindon, S. & Gascuel, O. A Simple, Fast, and Accurate Algorithm to Estimate Large Phylogenies by Maximum Likelihood. *Syst. Biol.* **52**, 696–704 (2003).
7. Francis, M., Lin, H., Rosa, J. C.-L., Doddapaneni, H. & Civerolo, E. L. Genome-based PCR Primers for Specific and Sensitive Detection and Quantification of *Xylella fastidiosa*. *Eur. J. Plant Pathol.* **115**, 203–213 (2006).
8. Nigro, F., Antelmi, I. & Ippolito, A. Identification and characterization of fungal species associated with the quick of olive. *Journal of Plant Pathology* **96**, S4.101-102 (2014).
9. Carlucci, A., Raimondo, M. L., Cibelli, F., Phillips, A. J. L. & Lops, F. *Pleurostomophora richardsiae*, *Neofusicoccum parvum* and *Phaeoacremonium aleophilum* associated with a decline of olives in southern Italy. *Phytopathol. Mediterr.* **52**, 517–527 (2013).
10. Suchard, M. A. *et al.* Bayesian phylogenetic and phylodynamic data integration using BEAST 1.10. *Virus Evol.* **4**, (2018).
