# Supplementary material for: Phylogenetic inference enables reconstruction of a long-overlooked outbreak of almond leaf scorch disease (Xylella fastidiosa) in Europe
Source: Commun Biol. 2020 Oct 9;3:560. doi: 10.1038/s42003-020-01284-7 (PMC7547738; doi:10.1038/s42003-020-01284-7)
Supplement: Supplementary file 2 — Description of Additional Supplementary Files [file 42003_2020_1284_MOESM2_ESM.pdf]

Description of Additional Supplementary Files

File Name: Supplementary Data 1
Description: Georeferenced data on ALSD incidence and mortality among orchards across Majorca island obtained in the field-assessment in 2017.

File Name: Supplementary Data 2
Description: Georeferenced data from almond orchards across Majorca island examined in Google street view to estimate the incidence and mortality of almond leaf scorch disease in 2012.

File Name: Supplementary Data 3
Description: Almond leaf scorch disease development sequence over time monitored through images form Google street view.

File Name: Supplementary Data 4
Description: Xf-DNA in growth rings

- Supplementary Data 4A. Dendrochronology + qPCR
- Supplementary Data 4B. Harper LOSVIB
- Supplementary Data 4C. Harper +Burbank (CSIC)
- Supplementary Data 4D. Survival analysis data

File Name: Supplementary Data 5
Description: Annual cumulative water deficit index between 1988 and 2017 from nine meteorological stations in Majorca at AEMET (aemet.es)

File Name: Supplementary Data 6
Description: List of genomes of Xylella used in this study, ST, origin, host plant and accession number.

File Name: Supplementary Software 1
Description: R script used in the statistical analyses and descriptive statistics with DnaSP6.
