# Supplementary material for: Phylogenetic inference enables reconstruction of a long-overlooked outbreak of almond leaf scorch disease (Xylella fastidiosa) in Europe
Source: Commun Biol. 2020 Oct 9;3:560. doi: 10.1038/s42003-020-01284-7 (PMC7547738; doi:10.1038/s42003-020-01284-7)
Supplement: Supplementary file 9 — Supplementary Software 1 [file 42003_2020_1284_MOESM9_ESM.docx]

**Supplementary Software 1**

*###################################*

*Disease incidence and mortality in 2017*

####################################

# We retrieved the data for the assessment from Supplementary Data 1

> ALSD2017<-read.csv2(Supplementary_Data_1)

> mean(ALSD2017$Incidence)

#To calculate the standard error of the mean (SEM)

> sd(ALSD2017$Incidence)/sqrt(length(ALSD2017$Incidence[!is.na(ALSD2017$ Incidence)]))

# To estimate the number of almond trees infected across Majorca

mean (ALSD2017$Incidence) *19417*81 *0.01

# Tree Mortality (2017 almond plantation census: 19,417 ha with an average density of 81 trees ha^-1^; https://www.mapama.gob.es)

> mean (ALSD2017$Mortality)*19417*81 *0.01

###########################################################

*Effect of tree age and distance on disease incidence and mortality in 2017*

###########################################################

# We retrieved the data for the assessment from Supplementary Data 1

#library(lme4)

mod<-glm(Incidence....~ Tree.age + Distance.from.Son.Carrió, data=ALSD2017)

> summary(mod)

glm(formula = Incidence.... ~ Tree.age + Distance.from.Son.Carrió, data = ALSD2017)

Deviance Residuals:

Min 1Q Median 3Q Max

-67.658 -7.658 2.342 13.868 27.693

Coefficients:

Estimate Std. Error t value Pr(>|t|)

(Intercept) 87.406 5.426 16.108 < 2e-16 ***

Tree.age>30 3.552 5.226 0.680 0.498

Distance.from.Son.Carrió>20km -18.651 3.715 -5.021 1.76e-06 ***

---

Signif. codes: 0 ‘***’ 0.001 ‘**’ 0.01 ‘*’ 0.05 ‘.’ 0.1 ‘ ’ 1

(Dispersion parameter for gaussian family taken to be 419.5953)

Null deviance: 62617 on 125 degrees of freedom

Residual deviance: 51610 on 123 degrees of freedom

AIC: 1123.5

Number of Fisher Scoring iterations: 2

> car::Anova(mod)

Analysis of Deviance Table (Type II tests)

Response: Incidence....

LR Chisq Df Pr(>Chisq)

Tree.age 0.4618 1 0.4968

Distance.from.Son.Carrió 25.2085 1 5.146e-07 ***

---

Signif. codes: 0 ‘***’ 0.001 ‘**’ 0.01 ‘*’ 0.05 ‘.’ 0.1 ‘ ’ 1

mod<-glm(Mortality....~ Tree.age + Distance.from.Son.Carrió, data=ALSD2017)

> summary(mod)

glm(formula = Mortality.... ~ Tree.age + Distance.from.Son.Carrió,

data = ALSD2017)

Deviance Residuals:

Min 1Q Median 3Q Max

-46.17 -18.34 -6.17 19.96 51.66

Coefficients:

Estimate Std. Error t value Pr(>|t|)

(Intercept) 36.095 5.995 6.020 1.86e-08 ***

Tree.age>30 13.375 5.775 2.316 0.0222 *

Distance.from.Son.Carrió>20km -21.126 4.104 -5.147 1.02e-06 ***

---

Signif. codes: 0 ‘***’ 0.001 ‘**’ 0.01 ‘*’ 0.05 ‘.’ 0.1 ‘ ’ 1

(Dispersion parameter for gaussian family taken to be 512.2506)

Null deviance: 80204 on 125 degrees of freedom

Residual deviance: 63007 on 23 degrees of freedom

AIC: 1148.6

Number of Fisher Scoring iterations: 2

> car::Anova(mod)

Analysis of Deviance Table (Type II tests)

Response: Mortality....

LR Chisq Df Pr(>Chisq)

Tree.age 5.365 1 0.02054 *

Distance.from.Son.Carrió 26.492 1 2.646e-07 ***

---

Signif. codes: 0 ‘***’ 0.001 ‘**’ 0.01 ‘*’ 0.05 ‘.’ 0.1 ‘ ’ 1

*################################*

*Disease incidence and mortality in 2012*

################################

# We retrieved the data for the assessment from Supplementary Data 2

> ALSD_2012<-read.csv2("D:/Sistema/Usuario/Desktop/ALSD_2012.csv")

> mean(ALSD_2012$Incidence)

> sd(ALSD_2012$Incidence)/sqrt(length(ALSD_2012$Incidence[!is.na(ALSD_2012$ Incidence)]))

###################################################################

*Spearman correlation between the incidence of ALSD and mortality of trees within orchards in 2017*

###################################################################

# We retrieved the data for the assessment from Supplementary Data 1

> ALSD_2017<-read.csv2("D:/Sistema/Usuario/Desktop/ALSD_2017.csv")

cor.test(ALSD2017$Incidence...., ALSD2017$Mortality...., method = "spearman")

Spearman's rank correlation rho

data: ALSD2017$Incidence.... and ALSD2017$Mortality....

S = 40703, p-value < 2.2e-16

alternative hypothesis: true rho is not equal to 0

sample estimates: rho 0.8779072

#################################################################

*Spearman correlation between the incidence of ALSD and mortality of trees within orchards in 2012*

#################################################################

# We retrieved the data for the assessment from Supplementary Data 2

> ALSD2012<-read.csv2("D:/Sistema/Usuario/Desktop/ALSD_2012.csv")

> cor.test(ALSD2012$incidencia, ALSD_2012$Severity, method = "spearman")

cor.test(ALSD2012$Incidence.., ALSD2012$Mortality...., method = "spearman")

Spearman's rank correlation rho

data: ALSD2012$Incidence.. and ALSD2012$Mortality....

S = 294700, p-value < 2.2e-16

alternative hypothesis: true rho is not equal to 0

sample estimates: rho 0.8854644

*####################################################*

*Friedman's ANOVA by ranks & Kendall's coefficient of agreement*

####################################################

# We retrieved the data for the assessment from Supplementary Data 3

> fried<-read.csv2("D:/Sistema/Usuario/Desktop/Friedman_rank.csv")

> head(fried)

Time 1 Time2 Time3 Time4 Time 5

1 1 2 4 4 NA

2 1 2 4 NA NA

3 1 2 3 4 NA

4 1 2 2 3 4

5 1 2 2 4 NA

6 1 2 4 NA NA

> fried1<-as.matrix(fried)

> friedman.test(fried1)

Friedman rank sum test

data: fried1

Friedman chi-squared = 42.411, df = 4, p-value = 1.371e-08

> library(irr)

Loading required package: lpSolve

> kendall(t(fried))

Kendall's coefficient of concordance W

Subjects = 3, Raters = 71, W = 0.696

Chisq(2) = 98.9

p-value = 3.35e-22

fried<-read.csv2("D:/Sistema/Usuario/Desktop/Friedman_rank.csv")

> str(fried)

'data.frame': 71 obs. of 5 variables:

$ tiempo1: int 1 1 1 1 1 1 1 1 1 1 ...

$ tiempo2: int 2 2 2 2 2 2 2 1 2 2 ...

$ tiempo3: int 4 4 3 2 2 4 2 2 3 3 ...

$ tiempo4: int 4 NA 4 3 4 NA 4 3 3 3 ...

$ tiempo5: int NA NA NA 4 NA NA NA NA NA NA ...

>fried<-fried%>% gather(key="time", value ="score", tiempo1,tiempo2,tiempo3,tiempo4,tiempo5)%>% convert_as_factor(id,time)head (fried,3)

fried<-fried%>% gather(key="time", value ="score", tiempo1,tiempo2,tiempo3,tiempo4,tiempo5)%>% convert_as_factor(id,time)head (fried,3)

ggboxplot(fried, x = "time", y = "score", add = "jitter")

##############

*Survival Analysis*

##############

# We retrieved the data for the assessment from Supplementary Data 4B

> survival3<-read.csv2("D:/Sistema/usuario/Desktop/survival3.csv")

> km <- with(survival3, Surv(Year, Censor))

> km_fit <- survfit(Surv(Year, Censor) ~ 1, data=survival3)

> summary(km_fit)

Call: survfit(formula = Surv(Year, Censor) ~ 1, data = survival3)

Time n.risk n.event survival std.err lower 95% CI upper 95% CI

1 30 1 0.967 0.0328 0.905 1.000

4 28 1 0.932 0.0463 0.846 1.000

5 27 1 0.898 0.0560 0.794 1.000

6 23 1 0.859 0.0658 0.739 0.998

8 20 2 0.773 0.0826 0.627 0.953

9 16 2 0.676 0.0965 0.511 0.894

11 11 1 0.615 0.1055 0.439 0.860

13 10 1 0.553 0.1114 0.373 0.821

14 9 2 0.430 0.1157 0.254 0.729

15 7 3 0.246 0.1042 0.107 0.564

16 4 2 0.123 0.0806 0.034 0.444

19 1 1 0.000 NaN NA NA

#Plot

> ggsurvplot(km_fit, data = survival3)

> ggsurvplot(km_fit, xlab="Year", risk.table=TRUE,data = survival3)

> ggsurvplot(km_fit, xlab="Year",surv.median.line = "hv",risk.table=TRUE,data = survival3)

> ggsurvplot(km_fit, xlab="Year",main= "Kepler-Meir Plot",surv.median.line = "hv",risk.table=TRUE,data = survival3)

> ggsurvplot(km_fit, xlab="Year", title= "Kepler-Meir Plot",surv.median.line = "hv",risk.table=TRUE,data = survival3)

*##################################*

*Differences of CWDi before and after 2003*

##################################

# We retrieved the data for the assessment from Supplementary Data 5

> period<-read.csv2("D:/Sistema/Usuario/Desktop/CWDi_period.csv")

> str(period)

'data.frame': 30 obs. of 3 variables:

$ Year : int 2017 2016 2015 2014 2013 2012 2011 2010 2009 2008 ...

$ CWDi : num -302 -226 -260 -304 -241 ...

$ Period: Factor w/ 2 levels "After 2003","Before 2003": 1 1 1 1 1 1 1 1 1 1 ...

t.test(WDI~Period, data=period,var.equal=TRUE,conf.level=0.95)

Two Sample t-test

data: CWDi by Period

t = 1.2509, df = 28, p-value = 0.2213

alternative hypothesis: true difference in means is not equal to 0

95 percent confidence interval:

-17.66441 73.07775

sample estimates:

mean in group After 2003 mean in group Before 2003

-235.6753 -263.3820

###########################

*Relationship between Cq and year*

###########################

#Linear relationship between Cq and Year. Data retrieved from Supplementary Data 4A

>pip<-read.csv2("D:/Sistema/Usuario/Desktop/Cq.csv")

str(pip)

'data.frame': 185 obs. of 2 variables:

$ Year: int 2017 2016 2014 2015 2012 2012 2012 2009 2008 2010 ...

$ Cq : num 33 33 34 33 34 34.5 36 36 37 36 ...

> mod0<-lm(Cq~Year, data=pip)

> mod0

lm(formula = Cq ~ Year, data = pip)

Coefficients:

(Intercept) Year

724.6181 -0.3448

> car::Anova(mod0)

Anova Table (Type II tests)

Response: Cq

Sum Sq Df F value Pr(>F)

Year 761.3 1 66.11 6.258e-14 ***

Residuals 2107.4 183

---

Signif. codes: 0 ‘***’ 0.001 ‘**’ 0.01 ‘*’ 0.05 ‘.’ 0.1 ‘ ’ 1

> cor(pip$Cq, pip$Year)

[1] -0.5151548

> summary(mod0)

lm(formula = Cq ~ Year, data = pip)

Residuals:

Min 1Q Median 3Q Max

-8.9958 -2.5135 0.4522 2.6938 5.8313

Coefficients:

Estimate Std. Error t value Pr(>|t|)

(Intercept) 724.61813 85.20714 8.504 6.37e-15 ***

Year -0.34479 0.04241 -8.131 6.26e-14 ***

---

Signif. codes: 0 ‘***’ 0.001 ‘**’ 0.01 ‘*’ 0.05 ‘.’ 0.1 ‘ ’ 1

Residual standard error: 3.393 on 183 degrees of freedom

Multiple R-squared: 0.2654, Adjusted R-squared: 0.2614

F-statistic: 66.11 on 1 and 183 DF p-value: 6.258e-14

################

Root-to-tip distances

################

*Xylella fastidiosa* subsp. *fastidiosa*

> roottotip<-read.csv2("D:/Sistema/Usuario/Desktop/rtp_fastidiosa.csv")

> rtp=lm(distance~date, data=roottotip)

> summary(rtp)

Call:

lm(formula = distance ~ date, data = roottotip)

Residuals:

Min 1Q Median 3Q Max

-3.246e-04 -4.741e-05 -3.891e-05 2.280e-05 6.144e-04

Coefficients:

Estimate Std. Error t value Pr(>|t|)

(Intercept) -4.136e-02 6.569e-03 -6.296 1.37e-06 ***

date 2.098e-05 3.273e-06 6.409 1.04e-06 ***

---

Signif. codes: 0 ‘***’ 0.001 ‘**’ 0.01 ‘*’ 0.05 ‘.’ 0.1 ‘ ’ 1

Residual standard error: 0.0002294 on 25 degrees of freedom

Multiple R-squared: 0.6216, Adjusted R-squared: 0.6065

F-statistic: 41.08 on 1 and 25 DF, p-value: 1.038e-06

> plot(distance~date, data=roottotip)

> abline(rtp)

*Xylella fastidiosa* subsp. *multiplex*

> read.csv2("D:/Sistema/Usuario/Desktop/rtip_multiplex.csv")

package ‘car’ was built under R version 3.5.3

> roottotip<-read.csv2("D:/Sistema/Usuario/Desktop/rtip_multiplex.csv")

> rtp=lm(distance~date, data=roottotip)

> summary(rtp)

Call:

lm(formula = distance ~ date, data = roottotip)

Residuals:

Min 1Q Median 3Q Max

-4.863e-04 -5.776e-05 -4.180e-05 2.820e-05 5.575e-04

Coefficients:

Estimate Std. Error t value Pr(>|t|)

(Intercept) -3.150e-02 1.411e-02 -2.232 0.0357 *

date 1.596e-05 7.007e-06 2.277 0.0324 *

---

Signif. codes: 0 ‘***’ 0.001 ‘**’ 0.01 ‘*’ 0.05 ‘.’ 0.1 ‘ ’ 1

Residual standard error: 0.0002145 on 23 degrees of freedom

Multiple R-squared: 0.184, Adjusted R-squared: 0.1485

F-statistic: 5.185 on 1 and 23 DF, p-value: 0.0324

> plot(distance~date, data=roottotip)

> abline(rtp)

> slope(rtp)

###########################

Descriptive statistics with DnaSP6

###########################

*Xylella fastidiosa* subsp. *fastidiosa*

DnaSP Ver. 6.12.03 27 - diciembre - 2019 20:52:18

==========================================================================

Polymorphic Sites

-----------------

Input Data File: D:\...\Fastidiosa27.phy

Number of sequences: 27 Number of sequences used: 27

Selected region: 1-1713597 Number of sites: 1713597

Total number of sites (excluding sites with gaps / missing data): 1657356

Sites with alignment gaps or missing data: 56241

Invariable (monomorphic) sites: 1655116

Variable (polymorphic) sites: 2239 (Total number of mutations: 2241)

Singleton variable sites: 839

Parsimony informative sites: 1401

Singleton variable sites (two variants): 839

Parsimony informative sites (two variants): 1400

Singleton variable sites (three variants): 0

Parsimony informative sites (three variants): 1

Variable sites (four variants): 0

Protein Coding Region assignation: No

--------------------------------

Gene Flow and Genetic Differentiation

-------------------------------------

Input Data File: D:\...\Fastidiosa27.phy

Number of Populations Included: 7

Selected region: 1-1713597 Number of sites: 1713597

Sites with alignment gaps are: Excluded

Total sites (excluding alignment gaps): 1657356

Population 1: Mallorcanclade

Number of sequences: 15

Nucleotide diversity, Pi: 0,00000

Population 2: USA

Number of sequences: 12

Nucleotide diversity with JC, PiJC: 0,00051

Population 5: CAL

Number of sequences: 5

Nucleotide diversity, Pi: 0,00022

--------------------

Input Data File: D:\...\Mallorcafastidiosa.phy

Selected region: 1-1713597 Number of sites: 1713597

Total sites (excluding alignment gaps): 1700429

Population 1: CLADE1

Number of sequences: 6

Nucleotide diversity, Pi(1): 0,00000

Population 2: CLADE_2

Number of sequences: 9

Nucleotide diversity, Pi(2): 0,00000

Total data:

Number of sequences: 15

Number of polymorphic sites: 16

Total number of mutations: 16

Average number of nucleotide differences, k: 3,067

Nucleotide diversity, Pi(t): 0,00000

Between populations:

Number of fixed differences: 0

Mutations polymorphic in population 1, but monomorphic in population 2: 4

Mutations polymorphic in population 2, but monomorphic in population 1: 10

Shared Mutations: 2

Average number of nucleotide differences between populations: 3,333

Average number of nuc. subs. per site between populations, Dxy: 0,00000

Number of net nuc. subs. per site between populations, Da: 0,00000

DnaSP Ver. 6.12.03 20 - enero - 2020 11:29:04

======================================================================

=========== Haplotype Distribution ===========

Number of haplotypes, h: 10

Haplotype diversity, Hd: 0,8952

Hap_1: 1 [1]

Hap_2: 1 [2]

Hap_3: 5 [3-4 11 14-15]

Hap_4: 1 [5]

Hap_5: 1 [6]

Hap_6: 2 [7 10]

Hap_7: 1 [8]

Hap_8: 1 [9]

Hap_9: 1 [12]

Hap_10: 1 [13]

Hap_1: 1 [28XfasIVIA]

Hap_2: 1 [44XfasXYL1]

Hap_3: 5 [45XfasXYL2 62Xy1978_2 70Xy2153_2 74Xy2508_2 76Xy3967_2]

Hap_4: 1 [63Xy1980_2]

Hap_5: 1 [65Xy2014_2]

Hap_6: 2 [66Xy2017_2 69Xy2107_2]

Hap_7: 1 [67Xy2093_2]

Hap_8: 1 [68Xy2106_2]

Hap_9: 1 [71Xy2177_2]

Hap_10: 1 [72Xy2400_2]

=========== Gene Flow Estimates ===========

Genome: Haploid

---------------------------------------------------------------------------------------

Population 1 Population 2 Fst

Mallorcanclade USA 0,43021

Mallorcanclade Mall2 0,00870

Mallorcanclade Mall1 -0,01551

Mallorcanclade CAL 0,11589

Mallorcanclade grapevines -0,08072

Mallorcanclade almond -0,08439

USA Mall2 0,43069

USA Mall1 0,43022

USA CAL 0,24859

USA grapevines 0,43021

USA almond 0,43014

Mall2 Mall1 0,21000

Mall2 CAL 0,11897

Mall2 grapevines 0,01923

Mall2 almond -0,01814

Mall1 CAL 0,11506

Mall1 grapevines -0,03571

Mall1 almond -0,02212

CAL grapevines 0,11563

CAL almond 0,11590

grapevines almond -0,04071

**-----------------------------------------------------------------------**

*Xylella fastidiosa* subsp*. multiplex*

Polymorphic Sites

-----------------

Input Data File: D:\...\concatenated25gMULTI_YYMM.nexus

Number of sequences: 25 Number of sequences used: 25

Selected region: 1-1615082 Number of sites: 1615082

Total number of sites (excluding sites with gaps / missing data): 1486735

Sites with alignment gaps or missing data: 128347

Invariable (monomorphic) sites: 1484224

Variable (polymorphic) sites: 2511 (Total number of mutations: 2526)

Singleton variable sites: 544

Parsimony informative sites: 1967

Singleton variable sites (two variants): 543

Parsimony informative sites (two variants): 1953

Singleton variable sites (three variants): 1

Parsimony informative sites (three variants): 14

Variable sites (four variants): 0

Protein Coding Region assignation: No

Overview: Polymorphism Data

---------------------------

Input Data File: D:\...\MALLORCA\Mallorca.phy

Number of sequences: 7 Number of sequences used: 7

Selected region: 1-1664716 Number of sites: 1664716

Total number of sites (excluding sites with gaps / missing data): 1651220

===================== G+C content =====================

G+C content, G+C: 0,529 (1651220,00 sites)

==== Analysis using the total number of positions ====

Number of variable sites, S: 11

Total number of mutations, Eta: 11

Nucleotide diversity (per site), Pi: 0,00000

Number of Haplotypes, h: 4

Gene Flow and Genetic Differentiation

-------------------------------------

Input Data File: D:\...\concatenated25gMULTI_YYMM.nexus

Number of Populations Included: 4

Selected region: 1-1615082 Number of sites: 1615082

Sites with alignment gaps are: Excluded

Total sites (excluding alignment gaps): 1486735

Population 1: CAL

Number of sequences: 6

Nucleotide diversity, Pi: 0,00041

Population 2: ALC

Number of sequences: 9

Nucleotide diversity, Pi: 0,00000

Population 3: Mallorca

Number of sequences: 7

Nucleotide diversity, Pi: 0,00000

Population 4: Corsica

Number of sequences: 3

Nucleotide diversity, Pi: 0,00000

Total Data Estimates

Number of sequences: 25

Number of segregating sites, S: 2511

Number of haplotypes, h: 18

Haplotype diversity, Hd: 0,95333

Average number of nucleotide differences, Kt: 616,32000

Nucleotide diversity, PiT: 0,00041

----------------------------------------------
