# Supplementary material for: Phylogenetic inference enables reconstruction of a long-overlooked outbreak of almond leaf scorch disease (Xylella fastidiosa) in Europe
Source: Commun Biol. 2020 Oct 9;3:560. doi: 10.1038/s42003-020-01284-7 (PMC7547738; doi:10.1038/s42003-020-01284-7)
Supplement: Supplementary file 11 — Reporting Summary [file 42003_2020_1284_MOESM11_ESM.pdf]

nature research

Corresponding author(s): Eduardo Moralejo

Last updated by author(s): Aug 27, 2020

# Reporting Summary

Nature Research wishes to improve the reproducibility of the work that we publish. This form provides structure for consistency and transparency in reporting. For further information on Nature Research policies, see our Editorial Policies and the Editorial Policy Checklist.

## Statistics

For all statistical analyses, confirm that the following items are present in the figure legend, table legend, main text, or Methods section.

| n/a | Confirmed | |
|---|---|---|
| ☐ | ☒ | The exact sample size (*n*) for each experimental group/condition, given as a discrete number and unit of measurement |
| ☐ | ☒ | A statement on whether measurements were taken from distinct samples or whether the same sample was measured repeatedly |
| ☐ | ☒ | The statistical test(s) used AND whether they are one- or two-sided<br>*Only common tests should be described solely by name; describe more complex techniques in the Methods section.* |
| ☒ | ☐ | A description of all covariates tested |
| ☒ | ☐ | A description of any assumptions or corrections, such as tests of normality and adjustment for multiple comparisons |
| ☐ | ☒ | A full description of the statistical parameters including central tendency (e.g. means) or other basic estimates (e.g. regression coefficient) AND variation (e.g. standard deviation) or associated estimates of uncertainty (e.g. confidence intervals) |
| ☐ | ☒ | For null hypothesis testing, the test statistic (e.g. *F*, *t*, *r*) with confidence intervals, effect sizes, degrees of freedom and *P* value noted<br>*Give P values as exact values whenever suitable.* |
| ☐ | ☒ | For Bayesian analysis, information on the choice of priors and Markov chain Monte Carlo settings |
| ☒ | ☐ | For hierarchical and complex designs, identification of the appropriate level for tests and full reporting of outcomes |
| ☐ | ☒ | Estimates of effect sizes (e.g. Cohen's *d*, Pearson's *r*), indicating how they were calculated |

*Our web collection on statistics for biologists contains articles on many of the points above.*

## Software and code

Policy information about availability of computer code

| | |
|---|---|
| Data collection | Almond leaf scorch incidence, severity and mortality for 2012 were estimated from images obtained from Google Street View (Details are given in Materials and Methods and Supplementary Information) |
| Data analysis | All sotware used is stated in the Material and Methods and Supplementary Information.<br>Statistic analyses were performed with R; two supplementary database were included as files in the submission.<br>R Development Core Team, R: A language and environment for statistical computing. Vienna, Austria (2017) https:/doi.org/R Foundation for Statistical Computing, Vienna, Austria. ISBN 3-900051-07-0, URL http://www.R-project.org.<br>Maps showing disease incidence and mortality distribution were constructed with QGIS from database1 and database 2.<br>QGIS Development Team, QGIS Geographic Information System. Open Source Geospatial Found. Proj. (2016).<br>BEAST: A. J. Drummond, M. A. Suchard, D. Xie, A. Rambaut, Bayesian phylogenetics with BEAUti and the BEAST 1.7. Mol. Biol. Evol. (2012)<br>PartitionFinder: R. Lanfear, P. B. Frandsen, A. M. Wright, T. Senfeld, B. Calcott, PartitionFinder 2: New Methods for Selecting Partitioned Models of Evolution for Molecular and Morphological Phylogenetic Analyses. Mol. Biol. Evol 34, 772–773 (2016). |

For manuscripts utilizing custom algorithms or software that are central to the research but not yet described in published literature, software must be made available to editors and reviewers. We strongly encourage code deposition in a community repository (e.g. GitHub). See the Nature Research guidelines for submitting code & software for further information.

## Data

Policy information about availability of data

All manuscripts must include a data availability statement. This statement should provide the following information, where applicable:

- Accession codes, unique identifiers, or web links for publicly available datasets
- A list of figures that have associated raw data
- A description of any restrictions on data availability

*Provide your data availability statement here.*

# Field-specific reporting

Please select the one below that is the best fit for your research. If you are not sure, read the appropriate sections before making your selection.

☐ Life sciences ☐ Behavioural & social sciences ☒ Ecological, evolutionary & environmental sciences

For a reference copy of the document with all sections, see nature.com/documents/nr-reporting-summary-flat.pdf

# Ecological, evolutionary & environmental sciences study design

All studies must disclose on these points even when the disclosure is negative.

| | |
|---|---|
| Study description | Full descriptions of the aims and logical reasoning for the each of the studies covering the epidemiology of ALSD are included in the Material & Methods subsections and in the Supplementary Information (disease incidence and mortality estimations, combination of dendrochronology and qPCR, survival analysis, temporal correlations between sequence of symptoms development, pathogenicity and transmission tests and climate data)<br>A second section includes the phylogenetic analyses using Bayesian inference to estimate the likely time of the most recent common ancestors of the two X. fastidiosa subspecies involved in the ALSD in Mallorca. |
| Research sample | Our research targets were the whole population of non-irrigated almond trees of Mallorca, either affected or not by ALSD, and the whole population of Xylella fastidiosa associated with the disease.<br><br>To estimate the disease incidence, severity and mortality in 2017, field observations were carried out during summer months (full details provided in Material & Methods). Database is georeferenced and provided in Supplementary Data 1,2 & 3 file. We tried to include observations distributed across the island to map the disease incidence and mortality spatial distribution. To link the relationship between almond decline attributed to fungi and ALSD symptoms caused by X. fastidiosa we explored historical images from Google Map View. We explain why we collected data from 2012 and how we estimated ALSD incidence and mortality within orchards in the Material & Methods sections. The data is included in Supplementary Data 2 file.<br><br>Almond varieties included in the pathogenicity tests were those available supplied from a nursery. We used a susceptible local variety Vivot for the transmission experiments.<br><br>Around June 2017 we realized that wood samples of infected almond trees contained X. fastidiosa DNA that could be easily detected by qPCR. We instructed personal involved in destroying infected tree (all almond trees that were positive to X. fastidiosa had to be removed following mandatory EU Decision) to collect disks for the dendrochronology analysis. Wood disks were taken from 34 felled trees in 2017 and 2018 distributed across the island. Raw data is available in Supplementary Data 4.<br><br>The number of isolates belonging to subsp. fastidiosa and multiplex from the Balearic Islands is a little unbalanced due to the known difficulties to obtain pure cultures of the subspecies multiplex. Nonetheless we included isolates from diverse localities and hosts to have an idea of the genetic diversity of both subspecies. |
| Sampling strategy | The logical reasoning for the sampling strategy are described in each section of the Material & Methods and Supplementary Information. |
| Data collection | All sample data have been collected, procesed and coded in the Official Laboratory of Plant Health from the Balearic Islands. The database is regularly checked and audited by the European Commission |
| Timing and spatial scale | Field observations, inoculation and transmission experiments, and isolations of X. fastidiosa from infected plant material were performed in the summers of 2017 and 2018. DNA extractions, qPCR, MLST and genome sequencing was carried out from November 2016 to November 2019.<br><br>The spatial scale is explained in the Material & Method. |
| Data exclusions | There were no data exclusion. |
| Reproducibility | We included replicates in the inoculation and transmission tests. |

| | |
|---|---|
| Randomization | To estimate the disease incidence, severity and mortalitysampling unit (orchards) consisted of 30 almond trees visually examined within three to five randomly selected rows. |
| Blinding | Blinding was not applied in the field data because we focus to gain a good distribution. |

Did the study involve field work? ☒ Yes ☐ No

## Field work, collection and transport

| | |
|---|---|
| Field conditions | Leaf samples were taken mostly in summer when the temperatures enhance the pathogen growth and symptoms are more visible. Relevant climatic data from 9 weather stations are included to estimate the effect of drought in the almond decline through time. |
| Location | All sample data have been collected and coded in the Official Laboratory of Plant Health from the Balearic Islands, which are regularly checked and audited by the European Commission. Latitude and longitude data are provided in Supplementary Data 1 and 2 files and under request. |
| Access & import/export | All sample processing, inoculation and transmission experiments hasve been performed at the Official Laboratory of the Plant Health department of the Balearic Islands (LOSVIB) in Mallorca and Government facilities in compliance with local, national and international laws. |
| Disturbance | All experiments were carried out with Xylella fastidiosa isolates recovered from the Balearic islands to minimize the risks of new introductions. |

# Reporting for specific materials, systems and methods

We require information from authors about some types of materials, experimental systems and methods used in many studies. Here, indicate whether each material, system or method listed is relevant to your study. If you are not sure if a list item applies to your research, read the appropriate section before selecting a response.

### Materials & experimental systems

| n/a | Involved in the study | |
|---|---|---|
| ☒ | ☐ | Antibodies |
| ☒ | ☐ | Eukaryotic cell lines |
| ☒ | ☐ | Palaeontology and archaeology |
| ☒ | ☐ | Animals and other organisms |
| ☒ | ☐ | Human research participants |
| ☒ | ☐ | Clinical data |
| ☒ | ☐ | Dual use research of concern |

### Methods

| n/a | Involved in the study | |
|---|---|---|
| ☒ | ☐ | ChIP-seq |
| ☒ | ☐ | Flow cytometry |
| ☒ | ☐ | MRI-based neuroimaging |
